# Gut Microbiota and Antibiotic Treatments for the Main Non-Oncologic Hepato-Biliary-Pancreatic Disorders

**DOI:** 10.3390/antibiotics12061068

**Published:** 2023-06-17

**Authors:** Federica Di Vincenzo, Alberto Nicoletti, Marcantonio Negri, Federica Vitale, Lorenzo Zileri Dal Verme, Antonio Gasbarrini, Francesca Romana Ponziani, Lucia Cerrito

**Affiliations:** 1 Internal Medicine and Gastroenterology, Fondazione Policlinico Universitario Agostino Gemelli IRCCS, 00168 Rome, Italy; 2 Dipartimento Universitario di Medicina e Chirurgia Traslazionale, Università Cattolica del Sacro Cuore, 00168 Rome, Italy

**Keywords:** gut microbiota, dysbiosis, leaky gut, antibiotics, liver cirrhosis, hepatic encephalopathy, spontaneous bacterial peritonitis, acute pancreatitis, primary sclerosing cholangitis, primary biliary cholangitis

## Abstract

The gut microbiota is a pivotal actor in the maintenance of the balance in the complex interconnections of hepato-biliary-pancreatic system. It has both metabolic and immunologic functions, with an influence on the homeostasis of the whole organism and on the pathogenesis of a wide range of diseases, from non-neoplastic ones to tumorigenesis. The continuous bidirectional metabolic communication between gut and hepato-pancreatic district, through bile ducts and portal vein, leads to a continuous interaction with translocated bacteria and their products. Chronic liver disease and pancreatic disorders can lead to reduced intestinal motility, decreased bile acid synthesis and intestinal immune dysfunction, determining a compositional and functional imbalance in gut microbiota (dysbiosis), with potentially harmful consequences on the host’s health. The modulation of the gut microbiota by antibiotics represents a pioneering challenge with striking future therapeutic opportunities, even in non-infectious diseases. In this setting, antibiotics are aimed at harmonizing gut microbial function and, sometimes, composition. A more targeted and specific approach should be the goal to pursue in the future, tailoring the treatment according to the type of microbiota modulation to be achieved and using combined strategies.

## 1. Introduction

Liver and pancreas are closely interconnected for both anatomic features and metabolic reasons. In fact, they take part in processes with crucial significance for the general homeostasis of the adult organism: the pancreas produces enzymes and polypeptides necessary for digestion and regulates glucose blood levels through insulin secretion; the liver takes part to lipid digestion and glucose release from glycogen reservoirs, can stock several nutrients, purifies the blood from toxic metabolites or drugs or even old or damaged blood cells and produces proteins for blood clotting, for the maintenance of oncotic pressure and for drugs or hormones transportation.

The biliary system and the portal-splenic-mesenteric vessels represent the highways linking the liver and the pancreas with the intestine and its complex microbial community (gut microbiota), whose role in the maintenance of human health and in the pathogenesis of several diseases is still under investigation and is continuously evolving. The concept of microbiota recently evolved in the more complex “microbiome”, that includes the genome of these microorganisms and the interactions with their environment. An imbalance in this intricate system can unleash an altered local immune response and can be involved in the pathogenesis of several diseases through inflammatory mediated mechanisms [1,2]. The aim of this review is to explore the state of the art regarding the application of antibiotic treatments in the main benign hepato-biliary-pancreatic disorders, with a special focus on their activity on host gut microbiota.

## 2. Liver and Gut Microbiota

In 1921, Hoefert described for the first time the presence of alterations in the gut microbiota of patients with chronic liver disease. Indeed, the gut microbiota, together with bacterial metabolites and bioproducts, and the reciprocal interaction with the local immune system influence the outcome of different liver diseases, establishing the “gut-liver axis” [3,4], that is the result of a continuous bidirectional anatomic connection between the gut and the liver through both the bile ducts and the intestinal blood derived from the portal vein, with a continuous bidirectional “metabolic” cooperation involving bile acids, hormones and products of nutrients digestion and absorption. In normal physiology, the liver is always interacting with bacteria translocated from the gut and their products, in a peculiar local immune environment favoring tolerance, since an intact intestinal epithelial barrier protects the liver from an excess of gut bacteria and their metabolites [5]. Bacterial products translocated from the gut reach the liver via portal circulation, which is approximately the 70% of the hepatic circulation, and the systemic circulation through the mesenteric lymph nodes and may induce an inflammatory response by the activation of Toll-like receptors (TLRs).

Dysbiosis is nowadays recognized as a hallmark of disease in cirrhotic patients: their gut microbiota shows a reduced richness, relative overexpression of pathogens and loss of some keystone taxa. Looking at phylum level, *Bacteroidetes* are decreased in favor of *Fusobacteria* and *Proteobacteria*, such as *Enterobacteriaceae* and *Pasteurellaceae*; looking at family, genus and species division, *Enterobacteriaceae*, *Streptococcaceae* and *Veillonellaceae* are increased in abundance, whereas *Lachnospiraceae*, *Ruminococcaceae*, *Clostridium clusters* XI and XIVab, lactic acid bacteria, *Bifidobacteria* and *Faecalibacterium prausnitzii* seem to be reduced [6,7,8,9,10,11,12]. Intestinal dysbiosis accompanies cirrhosis progression, worsening in decompensated patients in a vicious circle that perpetuates and amplifies liver damage [13,14].

Nevertheless, increased intestinal permeability and translocation of bacteria from the intestine has been described, especially through the mesenteric lymph nodes, and it is recognized as a major cause of some of the main complications of liver cirrhosis and portal hypertension: hepatic encephalopathy (HE) and spontaneous bacterial peritonitis (SBP) [6,15,16].

### 2.1. Hepatic Encephalopathy

#### 2.1.1. Gut Microbiota and Hepatic Encephalopathy: Towards the Gut–Liver–Brain Axis

HE is a central nervous system complication of liver cirrhosis, liver insufficiency or portosystemic shunting, that is characterized by a broad range of neuropsychiatric symptoms, varying from minor cognitive dysfunction to lethargy, depressed consciousness, disorientation and coma [17]. Depending on the severity of clinical manifestations, HE has been traditionally classified into overt HE (OHE), exhibiting clinically manifest neurological and psychiatric abnormalities such as flapping tremors, drowsiness or even coma and covert/minimal HE (MHE) or West Haven grade I HE, which presents with slight cognitive deficits in the executive functions, including working memory, psychomotor speed and response inhibition [18,19]. Data on the prevalence of HE vary depending on the definition of HE, the severity of the disease and the underlying cause. Among patients with cirrhosis, the prevalence of subclinical HE ranges between 20% and 80%, while that of OHE from 16% to 21% in decompensated cirrhosis [20,21].

HE significantly compromises the quality of life of both affected patients and their caregivers, and its onset is associated with high risk of recurrence, hospital admission and poor survival [22].

The exact pathogenesis of HE is complex and not clearly understood; it seems to be multifactorial, with ammonia, neuroinflammation and endotoxins being considered causative factors.

Cirrhosis has been notoriously associated with small intestinal bacterial overgrowth (SIBO) in approximately 48 to 73% of patients [23]. SIBO is determined by reduced intestinal motility, decreased bile acid synthesis, decreased gastric acid production and intestinal immune dysfunction, and it has been validated as a significant risk factor for MHE [13,24]. Particularly, hydrogen-producing SIBO (H-SIBO), rather than methane-producing SIBO (M-SIBO), has recently been associated with liver cirrhosis and covert HE [25].

Bajaj et al. demonstrated that the differences in fecal microbiota composition between healthy controls and cirrhotic patients were more pronounced when considering the presence or absence of HE [26]. Strikingly, they found that specific bacterial families such as *Alcaligenaceae*, *Porphyromonadaceae* and *Enterobacteriaceae* were strongly associated with both cognition and inflammation in HE. Specifically, *Alcaligenaceae* are able to produce ammonia by degradation of urea, thus explaining the correlation with cognitive impairment [26]. Moreover, the same group of authors found that MHE patients had higher abundances of *Enterococcus*, *Megasphaera*, *Burkholderia* and *Veillonella* and a reduced abundance of *Roseburia* in the gut mucosal microbiota. Notably, the composition of mucosal microbiota was significantly different from that of fecal microbiota, suggesting that adherence and overgrowth of pathogenic bacteria in the gut mucosal microbiota (due to alteration of bile acids metabolism and decreased production of anti-microbial peptides or mucins) could lead to bacterial translocation, resulting in inflammation [27].

Due to the lack of uniform criteria for the diagnosis of MHE, data on gut microbiota signatures of patients with MHE are highly variable and depend on the specific etiology of liver cirrhosis [11,26,28,29].

In patients with liver cirrhosis, the SIBO reduces the pool of secondary bile acids, immunoglobulin A levels and antimicrobial peptides, thus activating mucosal immune responses and determining intestinal inflammation and impaired intestinal epithelium integrity [30,31]. Moreover, SIBO downregulates tight-junction protein expression, resulting in increased intestinal permeability. This condition, referred to as “leaky gut”, facilitates the transfer of pathogenic bacteria and their metabolites from the intestinal lumen to the blood [32]. Translocated bacterial products and metabolites, named pathogen-associated molecular patterns (PAMPs), are transported to the liver through the portal vein. PAMPs are recognized by TLRs of macrophages and hepatic Kupffer cells and, through the nuclear factor kappa-light-chain-enhancer of activated B cells (NF-κB) signaling pathways, activate the production of pro-inflammatory cytokines, such as tumor necrosis factor-alpha (TNF-α), interleukins (ILs), interferons and chemokines like chemokine (C-C motif) ligand 20 (CCL20), chemokine (C-X-C motif) ligand 13 (CXCL13) and C-X3-C motif chemokine ligand 1 (CX3CL1) [33,34,35]. Previous studies in cirrhotic patients with MHE showed that higher abundances of *Enterobacteriaceae*, *Fusobacteriaceae* and *Veillonellaceae* were associated with higher serum concentrations of IL-2, IL-13 and IL-23, and these increased cytokines correlated with MHE severity [26]. The association between MHE severity and increased proinflammatory cytokines was independent of the severity of liver cirrhosis or ammonia levels [36].

**Figure 1:**
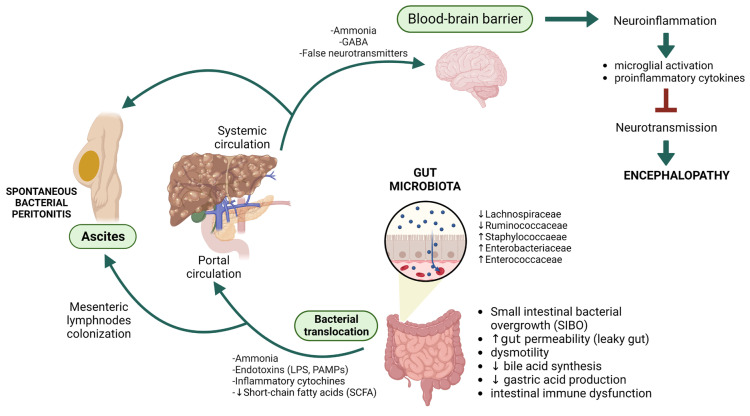
Gut microbiota is a pivotal actor in the complex interplay between gut and liver, concurring to the pathogenesis of liver cirrhosis complications such as hepatic encephalopathy and spontaneous bacterial peritonitis. Created with BioRender.com.

These cytokines, including interferon, TNF-α and ILs, downregulate tight junction proteins expression in the blood–brain barrier (BBB), alter BBB receptor expression and transport pathways, compromise cerebrovascular endothelial cells and activate astrocytes to an inflammatory reactive state. All these mechanisms impair the integrity and increase the permeability of BBB, thus determining a neuroinflammatory response in the brain to systemic inflammation [37,38]. Neuroinflammation is characterized by microglial activation and proinflammatory cytokine production in the cerebrum, interfering with neurotransmission, affecting neuronal functions and inducing low-grade cerebral edema in combination with hyperammonemia [39] (Figure 1).

Balzano et al. found that rats with chronic hyperammonemia and MHE experienced not only increased proinflammatory cytokines, such as IL-6, prostaglandin E2, IL-17 and TNF-α and reduced anti-inflammatory IL-10, but also microglial activation and increased messenger RiboNucleic Acid (mRNA) of IL-1β and TNF-α in the hippocampus of the rats, indicating the presence of both systemic and neuroinflammation in MHE [40]. Furthermore, cirrhotic mice models presenting dysbiosis with increased *Enterobacteriaceae* in the large intestine and increased *Staphylococcaceae*, *Lactobacillaceae* and *Streptococcaceae* in the small intestine experienced neuroinflammation and glial/microglial activation [41]. Germ-free mice colonized with feces from MHE patients containing increased levels of *Enterobacteriaceae*, *Staphylococcaceae* and *Streptococcaceae* had remarkable microglial activation, neuroinflammation and gamma-aminobutyric acid (GABA) signaling; reduction in neuroinflammation was observed using stool samples from the same patients after fecal microbiota transplant (FMT) from healthy donors, thus suggesting a direct effect of fecal microbiota on neuroinflammation and MHE, independently of active liver inflammation or injury [42].

Hyperammonemia is notoriously considered a causative agent of MHE; ammonia is derived from the degradation of aminoacids and urea by gut bacteria producing urease, especially Gram-negative *Enterobacteriaceae* [43]. Qin et al. found that most of the enteral consortia detectable in fecal samples of cirrhotic patients, such as *Streptococcus* spp. and *Veillonella* spp., belong to the oropharyngeal microbiota, thus suggesting an oralization of the gut bacterial environment [13]. Accordingly, Zhang et al. reported that in cirrhotic patients with MHE the fecal concentration of the gut urease-producing bacteria *Streptococcus salivarius* was significantly higher than in those without HE. Moreover, the amount of these bacteria positively correlated with hyperammonemia and ammonia accumulation [11]. Therefore, *Streptococcus salivarius* could be a potential therapeutic target for ammonia-lowering strategies in MHE patients. Hyperammonemia, similar to systemic inflammation, induces leaky BBB and promotes microglial activation, proinflammatory cytokines’ production and glutamine accumulation in the astrocytes, causing astrocyte swelling and low-grade cerebral edema that influences neurotransmission [44,45,46,47].

Another important actor in the development of MHE is endotoxemia. Cirrhotic patients with MHE are characterized by increased levels of *Veillonellaceae* and *Eubacteriaceae*, as well as increased endotoxemia, due to the presence of impaired intestinal barrier and portosystemic shunts [48,49]. Lipopolysaccharide (LPS) challenges in aged mice brain caused a hyperactivation of microglia and induction of mRNA expression of IL-1β, TNF-α, TLR-2 and IL-10, impairing glutamate transmission and resulting in memory and learning defects [50,51]. Accordingly, a study on mice models of acute liver failure showed that circulating LPS increased the level of proinflammatory cytokines and worsened liver pathology; moreover, LPS administration led to a rapid precipitation of hepatic coma in mice and cytotoxic brain swelling [52].

#### 2.1.2. Antibiotic Treatment for Overt Hepatic Encephalopathy

Current therapeutic strategies for the treatment of MHE in clinical practice are based on the modulation of the gut microbiota, in order to inhibit pathogenic bacteria overgrowth, modify gut microbiota composition and reduce the production and absorption of ammonia (Table 1). The frontline agent is lactulose, a synthetic non-absorbable disaccharide with the ability to reduce the time of contact between luminal contents and intestinal mucosa due to its cathartic effect, also lowering colonic pH creating a hostile environment for urease-producing bacteria, such as *Streptococcus salivarius*, and facilitating the growth of beneficial saccharolytic bacteria, such as *Bifidobacterium* and *Lactobacillus*, also reducing ammonia absorption by non-ionic diffusion [53].

The use of antibiotics is recommended as secondary prophylaxis in addition to lactulose. Neomycin, vancomycin, ampicillin and metronidazole are the main agents studied in the setting of OHE; unfortunately, side effects such as nephrotoxicity, ototoxicity and peripheral neuropathy limit their use in clinical practice, together with the risk of inducing bacterial resistance [54,55,56], while evidence on their efficacy on OHE are limited. For this reason, their long-term use is not recommended. Some clinical trials for the treatment of HE used nitazoxanide, a new broad-spectrum antibiotic and antiparasitic agent, with activity against anaerobes and with a good safety profile comparable to that of rifaximin; however, data on its efficacy are still lacking [57,58].

Rifaximin, an oral semisynthetic and minimally absorbed antibiotic directed against the β-subunit of bacterial RNA polymerase, with broad-spectrum activity against aerobic and anaerobic Gram-positive and Gram-negative bacteria, is the only recommended agent in this setting [59]. Rifaximin determined eubiotic modifications in the intestinal ecosystem, increasing *Bifidobacterium*, *Lactobacillus* and *Faecalibacterium prausnitzii*, and demonstrated anti-inflammatory properties, thus reducing bacterial virulence and microbial translocation from the gut [60].

The first study confirming rifaximin efficacy was published in 2010 by Bass et al. and consisted of a phase III, multicentric, randomized, double-blinded study versus placebo in cirrhotic patients with recurrent OHE, 90% of whom receiving also lactulose; rifaximin significantly reduced HE recurrence in a period of 6 months and the rate of hospitalizations, without significant differences between rifaximin and placebo regarding adverse events. The Food and Drug Administration (FDA) therefore approved the prescription of rifaximin for HE [61].

Subsequently, Eltawil et al. performed a metanalysis on 12 randomized controlled trials about HE treatment and observed that rifaximin effectiveness was equivalent to that of traditional oral antibiotics (neomycin or paromomycin) and non-absorbable disaccharide (lactulose or lactitol) but with a better safety profile. With regard to secondary outcomes, patients on rifaximin had significant improvement of electroencephalography and HE degree but also lower ammonia levels, asterixis reduction and better cognition, even if without reaching statistical significance [62].

In a recent study, Yokoyama et al. demonstrated that patients with H-SIBO had higher response-rates to rifaximin than those with M-SIBO [25]. Several studies revealed that rifaximin modulates gut microbiota function, by increasing serum levels of long-chain fatty acids and intermediates of carbohydrate metabolism and reducing serum proinflammatory cytokines, such as TNF-α, IL-1β and IL-6 [41,63]. Furthermore, rifaximin reduces endotoxemia and hyperammonemia generated through increased small-gut glutaminase activity and reduced intestinal glutamine levels, decreasing ammonia-producing bacteria, such as *Clostridium* and *Streptococcus* [64], and through the modulation of bacterial metabolites, such as LPS and secondary bile acids (deoxycholic acid) [41,65]. Regarding the gut microbiota composition, rifaximin determined only modest changes, such as reduction in *Streptococcus* and *Veillonella* abundance and increase in *Eubacteriaceae* [48,66,67]. Conversely, long-term treatment with rifaximin did not affect the gut microbiota composition in cirrhotic patients with MHE for over three months [68]. These results further support the hypothesis that rifaximin beneficial effects on MHE are mainly achieved by modulating the gut metabolome rather than the overall gut microbiota composition.

FMT is another therapeutic strategy, which has recently emerged for the treatment of HE, which is based on the transfer of fecal bacteria from a healthy donor to patients, in order to restore intestinal eubiosis [69]. In a randomized controlled trial, Bajaj et al. proved that FMT given after pre-treatment antibiotics (5 days of metronidazole, ciprofloxacin and amoxicillin) from a donor rationally selected based on the main gut microbiota alterations reported in patients with HE, thus high in *Lachnospiraceae* or *Ruminococcaceae*, reduced hospitalizations and improved cognitive function compared to standard of care in 20 cirrhotic patients suffering from recurrent OHE. Dysbiosis was also ameliorated after FMT, increasing beneficial taxa (*Lactobacillaceae* and *Bifidobacteriaceae*) and the relative abundance of *Lachnospiraceaeae*, compared to their post-antibiotic microbiome, and *Ruminococcaceae*, compared to baseline [70]. The same authors in a phase I trial using FMT via oral capsules without pre-treatment antibiotics, in recurrent OHE, showed the improvement of cognitive performances, increase in *Ruminococcaceae* and *Bifidobacteriaceae* and the reduction in *Streptococcaceae* and *Veillonellaceae* abundances post-FMT. Moreover, *Ruminococcaceae*, *Verrucomicrobiaceae* and *Lachnospiraceae* were linked with cognitive improvements and with decrease in inflammatory milieu after FMT. The FMT group showed reduced serum IL-6 and LPS-binding protein and higher deconjugation and secondary bile acids formation in serum and feces compared to baseline, whereas no change was seen in the placebo group. Notably, secondary bile acids were not increased in participants who developed poor outcomes, thus suggesting their role as biomarkers of poor outcomes [29,71]. Recently, a systematic meta-analysis comprising two randomized clinical trials, three case reports and three mice studies, for a total amount of 39 patients and 39 rodents, highlighted the association between FMT and improvement in neurocognitive tests, lower hospital readmission rate due to OHE episodes and reduction in serious adverse events [72] (Table 2).

Despite the potential benefits of FMT, uniform criteria for selecting donors, the optimal FMT dosing regimen and administration routes remain unclear. Moreover, due to the extreme frailty and vulnerability to infections of cirrhotic patients, rigorous screening, extended to the detection of antibiotic-resistant bacteria and careful selection of FMT donors would improve FMT safety.

Notably, FMT also proved to largely reduce the abundance of antibiotic resistance genes (ARGs) (beta-lactamase and vancomycin ARGs) in two different trials including cirrhotic patients treated, respectively, with one-time capsule FMT or placebo and with FMT by enema after pre-procedure with antibiotics compared to standard of care. Particularly, in the antibiotic and enema FMT group, there was a significant reduction in microbial DNA after the antibiotics, which could have promoted engraftment of the donor microbiota following FMT. In the enema FMT patients, there was an initial (day 7) increase in vancomycin and beta-lactamase ARGs, such as BlaZ beta-lactamase, after antibiotics, which subsequently decreased at day 15, whereas, compared with standard of care, at both day 7 and 15, the FMT patients had largely lower ARGs, such as CfxA beta-lactamase and vancomycin resistance genes. However, the use of pre procedure broad-spectrum antibiotics could have encouraged higher expression of quinolone and certain beta-lactamase genes that were incompletely reduced by subsequent FMT. On the other side, capsule-FMT induced a significant reduction in the preexisting LEN and OXY beta-lactamases genes post-FMT compared to baseline. Moreover, there was an increase in VanH expression in the placebo group over time, that was not present in the oral FMT patients [73].

### 2.2. Spontaneous Bacterial Peritonitis

#### 2.2.1. Spontaneous Bacterial Peritonitis and Intestinal Microbiota in Cirrhosis

Spontaneous bacterial peritonitis (SBP) is the most common infection seen in patients with advanced liver cirrhosis and ascites, being an important cause of acute decompensated cirrhosis, with a one-year mortality reaching 66% of patients [16]. It is a severe disease complication that requires rapid and accurate antibiotic treatment to improve clinical outcomes. However, due to absent/mild clinical symptoms in the early stage of SBP and the absence of noninvasive screening methods, it often deteriorates into acute on chronic liver failure (ACLF) and multi-organ failure, due to the late diagnosis and the lack of timely intervention [74]. Thus, SBP increases patients’ morbidity and mortality: despite advances in treatment, in-hospital mortality of patients with SBP reaches 25–30%. Nevertheless, recurrence of SBP, following the first episode, affects 69% of infected patients within one year [75].

The main risk factors for the development of SBP include high serum bilirubin levels, prior episodes of SBP, ascites protein levels <1g/dL and advanced liver disease [76]; cirrhotic patients with nucleotide-binding oligomerization domain containing 2 (NOD2) and TLR2 polymorphisms are at greater risk of developing SBP, showing a higher mortality than patients carrying minor alleles, together with an increased intestinal permeability and elevated markers of bacterial translocation [77,78]. Notably, in 50% of culture-based analysis of ascitic fluid, Gram-negative bacteria such as *Escherichia coli* and *Klebsiella* spp. as well as *Pneumococci*, *Streptococci* and other Gram-positive and Gram-negative bacteria have been identified [79]. However, bacterial DNA of intestinal microbes can be recognized in the ascitic fluid of half of cirrhotic patients even in absence of SBP and with negative cultures [6,7,8,80]. Further clinical and experimental investigations reported that the growth inhibition of intestinal Gram-negative aerobic flora reduces the incidence of SBP in cirrhotic patients [81]. Indeed, antibiotic prophylaxis is recommended by the American Association for Study of Liver Diseases [82] and European Association for the Study of the Liver guidelines [83,84] to prevent the development and recurrence of SBP [84,85]. However, evidence for the role and choice of antibiotics in both primary and secondary prophylaxis in the absence of gastrointestinal bleeding remains unclear.

The profound dysbiosis which characterizes liver cirrhosis is strictly linked to SBP.

A recent study by Zhou et al. demonstrated that patients with decompensated cirrhosis and SBP had a decreased microbial richness and increased microbial diversity [86]. Moreover, they found a different microbiota profile in patients with SBP compared with those without SBP, defined by an increase of 15 species including pathogens such as *Klebsiella pneumoniae*, *Serratia marcescens* and *Prevotella oris* and a decrease of some beneficial bacterial taxa, such as *Faecalibacterium prausnitzii*, *Methanobrevibacter smithii* and *Lactobacillus reuteri* [86]. Other studies revealed that *Enterobacteriaceae* are the most commonly translocated microbes identified in ascitic fluid of cirrhotic patients, and a higher proportion of Gram-negative bacteria in their gut microbiome was pointed out as the cause of SBP [8]. Notoriously, Gram-negative bacteria are the main etiological agents of SBP, with *K. pneumoniae* being the most common cause after *Escherichia coli* [84]. Therefore, these findings suggest that *K. pneumoniae* may translocate from the intestine to cause SBP. In cirrhotic patients with SBP, the abundance of the beneficial bacteria *Faecalibacterium prausnitzii* is negatively correlated with white blood cell count (WBC), C-reactive protein (CRP), procalcitonin (PCT) and Child–Turcotte–Pugh (CTP) score, while the abundance of *E. coli* was positively correlated with WBC, CTP score and Mayo End stage Liver Disease (MELD) score [86].

#### 2.2.2. Antibiotics and Spontaneous Bacterial Peritonitis

Considering the pathogenetic background, the evidence provides theoretical support for the preventive or therapeutic use of antibiotics in patients at risk for or who develop SBP [74,87] (Table 1 and Table 3).

Kalambokis et al. in 2012 showed that the administration of a 4-week regimen with rifaximin 1200 mg/day significantly reduced the neutrophil count in cirrhotic patients with sterile ascites, in association with a considerable decrease in plasma endotoxin [88]. Accordingly, in a prospective case–control study, the use of rifaximin significantly decreased the polymorpho-nuclear (PMN) cells count in ascitic fluid [89], and other data report a 72% reduction in the rate of SBP in cirrhotic patients with refractory ascites treated with rifaximin, compared with a control group [90]. Rifaximin seems also superior to norfloxacin in preventing bacterial translocation and SBP and is able to modulate blood pro-inflammatory and anti-inflammatory cytokines milieu in SBP patients [91]. Two systematic review and a meta-analysis, respectively, by Sidhu et al. [92] and Goel et al. [93] suggested a benefit for primary or secondary SBP prophylaxis in using rifaximin compared to norfloxacin. Accordingly, a recent network meta-analysis by Faust et al. of thirteen randomized controlled trials including 1742 patients revealed that rifaximin was superior to norfloxacin, as well as norfloxacin and trimethoprim-sulfamethoxazole to placebo, in reducing the rate of SBP. In addition, rifaximin ranked highest in reducing the risk of death/transplant [94]. Finally, the efficacy of rifaximin in association with norfloxacin has also been studied. Menshawy et al. in a meta-analysis including 973 patients showed that rifaximin in association with norfloxacin determined a reduced incidence of SBP than the norfloxacin-based regimen [95]. A prospective randomized open-label comparative multicenter study revealed that alternating norfloxacin and rifaximin for SBP primary prophylaxis showed higher efficacy, with the same safety profile as compared with norfloxacin monotherapy [96].

## 3. The Gut Microbiota in Acute Pancreatitis

Acute pancreatitis is a common cause of hospitalization around the world [97], with an increasing incidence in recent years [98]. Clinical manifestation may have different degrees of severity, as assessed by the 2013 modified Atlanta criteria, based on the presence of local complications and transient or persistent organ failure [99,100]. Acute pancreatitis can present as an edematous-interstitial form (80–85% of cases), which frequently resolves spontaneously in the absence of significant complications, or a necrotic-hemorrhagic form (15–20%) [101,102]. Acute necrotizing pancreatitis presents a significant risk for the development of acute/subacute complications, such as peripancreatic infected necrosis that is associated with a high mortality rate [103,104,105].

The role of the gut microbiota in acute pancreatitis has been evaluated in different studies [106], and possible pathogenic mechanisms have been hypothesized. Hypovolemia and consequent microcirculatory injury may cause intestinal mucosal ischemia, with reperfusion injury leading to intestinal barrier dysfunction and bacterial translocation [107].

Zhang et al. analyzed the heterogeneity in intestinal microbial composition in 45 patients with acute pancreatitis compared with 44 healthy controls using high-throughput 16S rRNA gene amplicon sequencing. The gut microbiota of patients with acute pancreatitis showed a reduced diversity and a remarkably different composition, with a higher abundance of *Proteobacteria* and *Bacteroidetes* and a depletion of *Firmicutes* and *Actinobacteria* compared to healthy individuals [108].

In another study, the DNA of *E. coli*, *Shigella* spp. and other intestinal bacteria was found in blood samples of patients with acute pancreatitis, confirming the hypothesis that intestinal opportunistic bacteria may enter the blood circulation through a damaged intestinal barrier, thus aggravating the progression of the disease and the occurrence of infectious complications [109].

The gut microbiota composition may also yield prognostic information. Yu et al. demonstrated that *Bacteroides* were predominant intestinal bacteria in mild acute pancreatitis, while *Escherichia* and *Shigella* were the most represented in moderately severe forms and *Enterococcus* in severe acute forms [110].

Similarly, Zhu et al. confirmed a general reduction in the gut microbiota diversity in patients with acute pancreatitis compared with healthy controls. At the phylum level, a reduction in *Bacteroidetes* and a relative abundance of *Proteobacteria* were observed. They also confirmed that *Escherichia* and *Shigella* were strongly associated with acute pancreatitis. Interestingly, the severity of acute pancreatitis correlated with gut microbiota dysbiosis in both human and animal models. Potentially harmful bacteria, such as *Acinetobacter* and *Stenotrophomonas*, were more abundant in the severe disease group. The abundance of *Escherichia* genera was associated with a higher rate of *Escherichia*-associated infection, confirming a role of intestinal permeability as a driver of complications in acute pancreatitis [111].

All these findings highlight the potential role of the gut microbiota as a driver of the complications of acute pancreatitis and as an additional element that could help stratify patient’s risk; they also pave the way for the identification of possible gut microbiota-based treatment targets. In fact, in a murine model, *Parabacteroides* could reduce neutrophilic infiltration in acute pancreatitis through the production of acetate, which enhances host defense against inflammation lowering neutrophils blood count resulting in less neutrophil infiltration in the pancreas [112].

In conclusion, further studies are needed to evaluate the causative influence of the gut microbiota in the development of acute pancreatitis and its complications. These results may help to develop personalized strategies for the treatment of the disease.

### Antibiotics in Acute Pancreatitis

Severe acute pancreatitis is the paradigm of systemic inflammatory response syndrome (SIRS), which can be triggered by either infectious or non-infectious proinflammatory noxa. Hence, the clinical presentation of both infected and non-infected necrotizing pancreatitis can be extremely similar. This has led to a significant overuse of antibiotics [113], which instead should be prescribed only when there is clear evidence of infected necrosis, such as gas in peripancreatic collections or positive culture of the bioptic material collected by fine needle aspiration (FNA) imaging signs, or when clear worsening or no clinical improvement is noted after 7–10 days of hospitalization according to SIRS indicators or Apache II score [114,115,116]. However, it is important to collect blood cultures in order to identify pathogens and guide targeted antibiotic therapy. Other clear indications for antibiotic therapy in the context of severe acute pancreatitis are associated cholecystitis and cholangitis [114].

Beyond clinical situations in which the indication for antibiotic treatment is clear, there are gray areas in clinical practice. In a meta-analysis of 31 different observational studies, advanced age, biliary etiology of acute pancreatitis, more than 50% pancreatic necrosis, delay in the resumption of enteral nutrition, presence of multiple organ failure or persistent multiorgan failure and the need for mechanical ventilation support were moderately to highly associated with an increased risk of infected pancreatic necrosis [117]. Similarly, in another meta-analysis elevated serum levels of LPS, C-reactive protein, PCT and an elevated Apache Score II were risk factors and predictors for the development of infected pancreatic necrosis [118] (Table 4).

A survey conducted on the use of antibiotics in Great Britain showed how the appropriateness of treatment is often inadequate and how the indication for antibiotic therapy in acute pancreatitis is correct only in a very small percentage of cases [119]. Similarly, Baltatzis et al. demonstrated that the use of antibiotics, both as prophylaxis and as treatment of acute pancreatitis is widespread, especially in mild forms. This overuse not only does not provide clinical benefit but also increases the risk of harm [120]. Therefore, the application of antibiotic stewardship programs to guide the responsible and optimal use of antibiotics would reduce side effects and costs. PCT-based algorithms are useful tools to reduce the prescription of antibiotics, although they are not validated for the detection of overt infection. Most clinicians initiate antibiotic therapy are based on increased WBC count and/or elevated C-Reactive-Protein (CRP), lipase and amylase levels, which however did not show association with infection in the early phase of acute pancreatitis. On the other hand, PCT levels proved to be a better biomarker of early infection [113]. In a single-center, patient-blinded, randomized controlled clinical trial by Siriwardena et al., patients were randomly assigned to a PCT-guided care group or a usual care group: 45% of them in the first group and 63% in the second group (63%) received antibiotics, with no significant difference between groups in terms of the number of infections, adverse events and mortality between the groups [121]. PCT-based algorithms are useful tools to reduce the prescription of antibiotics, although they are not validated for the detection of overt infection.

To make a correct choice of empiric antibiotic therapy, it must be considered that pathogens usually reach the pancreas from the blood, the biliary system, ascending from the duodenum via the main pancreatic duct or by transmural migration. For these reasons, the microorganisms most frequently involved in infected necrotizing pancreatitis are Gram-negative bacteria [122]. Antibiotics should have a good penetration into pancreatic necrotic tissue and present an adequate coverage spectrum. Normally, aminoglycosides are unable to reach concentrations sufficient to cover the minimal inhibitory concentration (MIC) of bacteria involved in secondary pancreatic infections, so they are not usually chosen as empirical treatment in acute pancreatitis [123]. Instead, the penetration of third-generation cephalosporins is intermediate, with a good efficacy on Gram-negative microorganisms; piperacillin/tazobactam has a broader coverage spectrum [124], while quinolones in association with metronidazole offer a good balance between penetration and antibacterial effect. Finally, carbapenems have the best penetration and coverage spectrum. However, they should be used only in the most critical patients in relation to the high and growing rate of resistance worldwide [125,126].

Antibiotic therapy alone can be an adequate treatment for infected necrotizing pancreatitis, as the POINTER trial demonstrated no benefit in the early drainage of infected necrosis. Indeed, the Comprehensive Complication Index was 57 in the immediate-drainage group vs. 58 in the postponed-drainage group (95% confidence interval (CI), −12–10; *p* = 0.90); mortality was 13% in the immediate-drainage group vs. 10% in the postponed-drainage group (relative risk 1.25; 95% CI, 0.42–3.68). Interestingly, 39% of the patients in the postponed-drainage group only received antibiotic therapy and did not require any drainage, with an excellent survival rate (89%, 17/19 patients). Patients in the postponed-drainage group underwent fewer interventional procedures (mean 2.6 vs. 4.4) [127].

Several studies and meta-analysis failed to demonstrate any benefit from antibiotic prophylaxis in acute pancreatitis [128,129,130,131,132]. Hence, main international guidelines do not recommend prophylactic antibiotic therapy in acute pancreatitis [114,116,133]. However, antibiotic prophylaxis has been a common prescription in acute pancreatitis, from mild to severe forms, for years [134,135,136,137]; only recently a clear recommendation against its use has favored a mild decline in the rate of prescriptions [138]. Although fungal infections, particularly *Candida albicans*, are associated with a higher risk of morbidity and mortality, there is currently no consistent evidence for the use of prophylactic antifungals [130,139,140,141].

Further studies are needed to investigate the effective role of selective intestinal decontamination in preventing superinfections [114].

## 4. Gut Microbiota and Antibiotics in Biliary Diseases

Acute biliary tract infections, such as acute cholecystitis and cholangitis, are common intra-abdominal infections. Since they present with peculiar clinical and imaging features, the indication for antibiotics is clear in most cases. Several international guidelines have been developed to guide the treatment of these conditions [126,142,143].

### 4.1. Primary Sclerosing Cholangitis

Primary sclerosing cholangitis (PSC) is an immune-mediated chronic cholestatic liver disease, whose characteristics are inflammation, fibrosis and destruction of intra and extrahepatic bile ducts which can lead to liver cirrhosis. In most cases, it is associated with inflammatory bowel diseases. Ductal inflammation can cause strictures in the common bile ducts and larger intrahepatic ducts that obstruct the bile flow, favoring superinfections. Hence, PSC with strictures require frequent use of antibiotics due to cholangitis [144,145]. The treatment of dominant biliary strictures is dilation by endoscopic retrograde cholangiopancreatography (ERCP). Due to the high risk of biliary contamination and superinfection during the procedure, antibiotic prophylaxis is recommended if complete drainage is unlikely or if the patient has cirrhosis and ascites [146,147,148,149].

The pathogenesis of PSC is not completely understood. However, recent studies hypothesized a role of a deranged gut–liver axis. Particularly, gut dysbiosis and increased intestinal permeability may be responsible for bacterial translocation of gut pathogens and detrimental microbial metabolites that may cause liver inflammation [150,151]. The analysis of the gut microbiota of patients with PSC showed an increased relative abundance in *Veillonella* genus, particularly at advanced stages with a positive correlation with the PSC Mayo score [152]. In another study, a relationship between *Veillonella* and *Fusobacterium* absolute abundance and intestinal inflammation (in terms of levels of fecal calprotectin) was observed, whereas the abundance of *Enterococcus* correlated with levels of alkaline phosphatase [153].

Dysbiosis in the biliary resident microbiota has also been considered a potential pathogenic driver and target for treatments [154]. *Veillonella*, *Streptococcus* and *Enterococcus* were found in the bile of patients with PSC [155,156].

Based on this assumption, some studies have been conducted to evaluate the use of antibiotics in the management of PSC with controversial results. Non-absorbable antibiotics, such as rifaximin, paromomycin, neomycin and oral vancomycin, were used with the primary aim of reducing the inflammatory elements and potential pathogens in the gut microbiota. Instead, absorbable antibiotics, such as metronidazole, cross the intestinal barrier to achieve serum and biliary therapeutic concentrations, thus acting in both the gut and bile.

The use of rifaximin showed no benefit on alkaline phosphatase (ALP) levels and other biochemical outcomes in a clinical trial including 16 patients with PSC [157]. In another open-label study, 16 patients with PSC were treated for 12 months with minocycline; a significant decrease in ALP was noticed, but 25% of patients withdrew from the study due to drug intolerance [158].

In a study by Färkkilä et al. [159], 80 patients were randomized to 36 months of ursodeoxycholic acid (UDCA) (15 mg/Kg/day) with metronidazole or UDCA alone. A significant reduction in ALP, Mayo risk score and histologic stage and grade was reported in the group treated with metronidazole and UDCA.

The most important data for clinical practice are derived from studies on vancomycin. A double-blind, randomized, pilot study was conducted in 35 patients that were randomized in four groups: low-dose vancomycin (125 mg four times daily), high-dose vancomycin (250 mg four times daily), low-dose metronidazole (250 mg three times daily) and high-dose metronidazole (500 mg three times daily). Low-dose and high-dose vancomycin groups were superior to metronidazole in achieving a significant reduction in ALP. The Mayo risk score decreased both in the low-dose vancomycin and low-dose metronidazole groups [160]. Similar results were observed in the treatment group of another study including 29 patients with PSC treated with UDCA that were randomized to vancomycin (125 mg qid) or placebo. The use of vancomycin was also associated with a reduction in pruritus, anorexia, fatigue and diarrhea [161].

In two open-label clinical trials with higher doses of vancomycin, a significant decrease of ALP, GGT and ALT was observed in the vast majority of patients (96%, 81% and 95%, respectively). Moreover, a significant number of patients experienced a normalization of these parameters (39%, 22% and 56%, respectively) within the first 6 months of therapy [162].

Table 5 summarizes the characteristics of the main studies about antibiotic treatment in patients with primary sclerosing cholangitis.

Finally, a meta-analysis demonstrated a beneficial effect of antibiotics on the reduction of ALP, serum bilirubin and Mayo risk score (33%, 29% and 36%, respectively) [163].

In conclusion, the use of antibiotics may play a role in the management of PSC. However, the ideal regimen, dosage and duration of antibiotic treatment in PSC are still unknown. Most of the trials were designed as pilot studies and set biochemical parameters as primary outcome. Hence, larger number of patients, the use of histopathology and clinical outcomes and longer follow-up periods are needed in order to confirm these promising results.

As for FMT, only one open-label pilot study including 10 patients with PSC was performed. No adverse events and an increase in the diversity of the microbiota were observed. However, the exiguous number of patients is insufficient to draw conclusions on the efficacy of this treatment [164].

**Table 5:** Characteristics and results of the studies about antibiotic treatment in patients with primary sclerosing cholangitis.

AUTHOR OF THE STUDY	STUDY DESIGN	STUDY POPULATION	AIM OF THE STUDY	PRIMARY ENDPOINTS	SECONDARY ENDPOINTS	RESULTS (% Change from Baseline Post-Therapy)	
Tabibian et al. (2017) [157]	12-week open-label pilot study	16 patients with PSC. 13 M and 3 F, median age 40 years old, 81% with IBD	Efficacy and safety of oral rifaximin 550 mg twice daily	Serum ALP at 12 weeks	Serum bilirubin, γ GT, PSC MRS at 12 weeks	ALP (+3.00—*p* = 0.47) MRS (+0.15—*p* = 0.21)	
Silveira et al. (2009) [158]	1-year pilot study	16 patients with PSC, gender non specified, median age 50 years old, 88% with IBD	Safety and efficacy of Minocycline 100 mg orally twice daily	Serum ALP at 1 year	PSC MRS at 1 year	ALP (−65—*p* = 0.04) MRS (−0.53—*p* = 0.05)	
Färkkilä et al. (2004) [159]	36-year multicenter, randomized, double-blind, placebo-controlled trial	80 patients with PSC (41 placebo, 39 MTZ), 42 M and 38 F, median age 16–65 years old, 81% with IBD	Effect of Metronidazole 800 mg compared with placebo on the progression of PSC	Serum ALP at 36 months	PSC MRS at 36 months	Metronidazole: ALP (−337—*p* = 0.05) MRS (−0.32—*p* = 0.05)	Placebo: ALP (−214—*p* < 0.01) MRS (−0.06—*p* < 0.01)
Rahimpour et al. (2016) [161]	Triple blinded, randomized, placebo-controlled trial	29 patients with PSC (11 placebo, 18 vancomycin), 17 M and 12 F, median age 36 years old, 75% with IBD	Safety and efficacy of oral Vancomycin (125 mg, four times a day)	ALP levels and the PSC MRS at 12 weeks	Serum level of ESR, AST, ALT, bilirubin, WBC, PLT, γ GT and symptoms at 12 weeks	ALP (−519.68—*p* = 0.11) Bilirubin (−1.35—*p* = 0.41) MRS (−0.59—*p* = 0.03)	
Tabibian et al. (2013) [160]	12-week randomized clinical trial	35 patients with PSC: 8 Vancomycin 125 mg/24 h 9 Vancomycin 250 mg/6 h 9 Metronidazole 250 mg/8 h 9 Metronidazole 500 mg/8 h (21 males and 14 females, median age 40 years old, 71% with IBD)	Safety and efficacy of oral Vancomycin and Metronidazole in patients with PSC	Serum ALP at 12 weeks	Serum bilirubin, PSC MRS, pruritus, adverse effects at 12 weeks	Vancomycin low-dose ALP (−188—*p* = 0.03) Bilirubin (−0.3—*p* = 0.06) MRS (−0.55—*p* = 0.03) Metronidazole low-dose ALP (+46—*p* = 0.47) Bilirubin (−0.2—*p* = 0.03) MRS (−0.16—*p* = 0.03)	Vancomycin high-dose ALP (−136—*p* = 0.02) Bilirubin (0—*p* = 0.48) MRS (−0.03—*p* = 0.98) Metronidazole high-dose ALP (−138—*p* = 0.22) Bilirubin (0.1—*p* = 0.78) MRS (−0.28—*p* = 0.16)
Ali et al. (2020) [162]	Open-label clinical trial	59 patients with PSC, 38 M and 21 F, median age 13.5 years old, 95% with IBD	Safety and efficacy of oral Vancomycin in patients with PSC	Decrease of ALP, γ GT and ALT from baseline	Not specified	ALP 81.3% γ GT 96% ALT 94.9%	
Deneau et al. (2018) [165]	Retrospective Study (data from Pediatric Consortium)	264 patients with PSC: 88 oral vancomicine (66 M and 22 F, median age 14 years old, 86% with IBD), 88 UCDA (72 M and 16 F, median age 12 years old, 85% with IBD), 88 observation (69 M and 19 F, median age 14 years old, 86% with IBD)	Safety and efficacy of oral Vancomycin and UCDA in patients with PSC	Serum γ GT < 50 U/L or ≥75% less than the pretreatment serum γ GT at 1 year	Improvement of liver fibrosis staging	Oral Vancomicine γ GT 53% (*p* = 0.918) Fibrosis 20% (*p* = 0.193) UCDA γ GT 49% (*p* = 0.918) Fibrosis 13% (*p* = 0.193) Observation γ GT 52% (*p* = 0.918) Fibrosis 18% (*p* = 0.193)	
Davies et al. (2008) [166]	Retrospective Study (data from Pediatric Consortium)	14 patients with PSC M/F 2.3:1 median age 12 years old 100% with IBD	Safety and efficacy of oral Vancomycin in patients with PSC	Serum γ GT < 50 U/L or ≥75% less than the pretreatment serum γ GT at 1 year	Improvement of liver fibrosis staging	Oral Vancomicine γ GT 53% (*p* = 0.918) Fibrosis 20% (*p* = 0.193) UCDA γ GT 49% (*p* = 0.918) Fibrosis 13% (*p* = 0.193) Observation γ GT 52% (*p* = 0.918) Fibrosis 18% (*p* = 0.193)	

Abbreviations: ALP, alkaline phosphatase; ALT, alanine aminotransferase; AST, aspartate aminotransferase; ESR, erythrocyte sedimentation rate; F, female; IBD, inflammatory bowel disease; γ GT, glutamyl transpeptidase; M = male; MRS, Mayo Risk Score; PLT, platelet; PSC, primary sclerosing cholangitis; WBC, white blood cells.

### 4.2. Primary Biliary Cholangitis

Primary biliary cholangitis (PBC) is a biliary autoimmune disease, characterized by a T-lymphocyte-mediated attack on small intralobular bile ducts. The chronic damage on the bile duct epithelial cells leads to their gradual destruction and eventual disappearance. The sustained loss of intralobular bile ducts causes the signs and symptoms of chronic cholestasis and eventually may result in cirrhosis and liver failure [167]. With the advent of ursodeoxycholic acid and later obeticholic acid, the majority of patients have normal life expectancy, and cirrhosis occurs in a minority of patients [168].

The diagnosis can be formulated on the basis of elevated alkaline phosphatase and presence of antimitochondrial antibodies (AMAs). AMAs can be found in 95% of PBC patients, and the majority of AMA-negative PBC patients have specific antinuclear antibodies. Therefore, diagnostic liver biopsy is no longer needed for the majority of patients [169,170].

Bacterial infections are an important risk factor for developing PBC, especially in female patients [171]. Molecular mimicry and immunological cross-reactivity between several bacteria and human mitochondrial antigens seem to be extremely important in the pathogenesis of PBC. The specific AMAs are directed against members of the 2-oxo-acid dehydrogenase complex family of enzymes. In over 95% of patients with PBC, pyruvate dehydrogenase complex E2 subunit (PDC-E2), an enzymatic complex that is expressed in mitochondria, or other proteins that share lipoic acid residues are the culprit autoantigens [172,173]. PDC-E2 shows cross-reactivity with several bacterial proteins, such as pyruvate dehydrogenase complex, ATP-dependent Clp protease, dihydrolipoamide acetyltransferase (E2p) and other proteins of *Escherichia coli*, lipoyl domains of *Novosphingobium aromaticivorans*, heat shock proteins of *Mycobacterium gordonae*, pyruvate dehydrogenase complex of *Mycoplasma pneumoniae* and β-galactosidase of *Lactobacillus delbrueckii*. Hence, an immune reaction against one or more of these bacteria, combined with a loss of immunotolerance to PDC- E2, may lead to the development of PBC [173,174]. The ubiquitous bacterium *Novosphingobium aromaticivorans* is the main bacterial candidate in the pathogenesis of PBC. In fact, two of its proteins showed a high degree of homology with the dominant immunogenic domain of the PDC-E2, representing the highest level of homology between this mitochondrial autoantigen and any known microorganism. In a study, sera from 77 out of 77 PBC patients (100%) reacted against the investigated bacterial proteins (at least 100-fold higher than the reactivity against Escherichia coli), whereas none of the 195 control sera showed reactivity towards the same antigens. Hence, *Novosphingobium aromaticivorans* might break tolerance to self PDC-E2 by two independent mechanisms, including alteration of bacterial PDC-E2 or host PDC-E2 by xenobiotics metabolism [175].

Furthermore, PBC seems to occur more frequently in patients with urinary tract infections, particularly by *Escherichia coli* or other infections by *Mycobacteria*, *Chlamydia* and *Helicobacter pylori* [176,177,178,179,180]. Elevated antibodies titers against *Enterobacteriaceae*, *Toxoplasma gondii* and *Helicobacter pylori* have also been reported [181].

The study of the gut microbiota by 16S RNA sequencing-based has identified the changes of the microbiota composition that are associated with PBC. In a study by Lv et al. [182], the gut microbiota of patients with early stage PBC showed a higher abundance of potentially opportunistic pathogens, such as the families *Enterobacteriaceae*, *Neisseriaceae* and *Enterococcaceae* and the genera *Streptococcus*, *Veillonella* and *Haemophilus parainfluenzae*, compared with healthy controls. Simultaneously, a decreased abundance in health-promoting bacteria, such as *Lachnospiraceae* and some beneficial *Bacteroidetes*, was observed.

Significant alterations of circulating bile acids in treatment naïve PBC patients are strongly associated with disease progression. PBC patients showed gut dysbiosis that correlated with the bile acid profile compared with healthy controls. UDCA treatment reversed the bile acid profile and dysbiosis in PBC patients. Thus, bile acid profiling may contribute to PBC patients’ diagnosis and disease status assessment. Altering the gut microbiota might allow modulation of the bile acid profile and, subsequently, be harnessed for PBC patients’ treatment [183].

Unlike PSC, there are only a few older studies on antibiotic treatment for PBC. The main reason is that therapies that modulate bile acids showed significant clinical benefit in the majority of patients. Moreover, the lack of high-risk individuals and the uncertainty on the initial steps of pathogenesis have not allowed to hypothesize preventive strategies by antibiotics or modulating the gut microbiota. Finally, PBC is limited to the small bile ducts of the liver, where the colonization of bacteria is still considered debatable [184].

The only antibiotic therapy that is commonly used in clinical practice is rifampicin, a heterocyclic antibiotic used as a second line for pruritus. Indeed, rifampicin activates the pregnane X receptor leading to decrease in autotaxin levels. The autotaxin enzyme synthesizes lysophosphatidic acid (LPA) which in turn activates TRP vanilloid 1 (TRPV1), a capsaicin receptor involved in the sensory transmission of itch [185].

At present, there is no evidence of the use of FMT in PBC.

## 5. Conclusions

The hepato-biliary-pancreatic system is composed of organs with closely interconnected functions, as in a complex machinery whose pivot is the gut microbiota. Indeed, our intestinal endobiome exerts metabolic and immunologic functions that impact on both the homeostasis of the whole organisms and also condition the onset of a wide range of non-communicable diseases, from non-neoplastic ones to tumorigenesis.

However, antibiotics are a double-edged sword, as the gut microbiota is also a potential target that can be influenced in either a positive or a negative way; in the latter case, even short-term antibiotic treatments lead to a compositional and functional imbalance, called dysbiosis, with potential harmful consequences on host’s health due to the impoverishment of the taxonomic composition of both luminal and mucosal gut microbiota composition and function [186] and unpredictably long-lasting effects [187,188]. Moreover, a reduction was observed in the resistance against the colonization by pathogen microorganisms due to the damage on the structure of intestinal mucus layer and subsequent alteration of the complex mutualistic host–microbiota interaction in the creation of gastrointestinal barrier [187,189,190,191].

The imbalance between the different species of gut microbiota can lead to the flourishing of pathogenic bacteria such as *Clostridium difficile*, which is responsible for severe infectious colitis (burdened by significant morbidity and mortality) [192], or to the selection of multi-drug resistant bacteria (e.g., vancomycin-resistant *Enterococci*, extended-spectrum beta-lactamase-carrying strains and carbapenemase-producing *Enterobacteria*) that represent a serious problem for public health and result in dramatically increasing patient’s mortality even due to their ability in transferring antibiotic resistance genes in commensal microbes [193,194] or the upregulation of resistance genes involving also non-administered antibiotic classes [195,196].

It is unavoidable that modifications produced by antibiotics on gut microbiota could perturb the complex cooperation between host and gut microbiota [197], and it is essential to restore the lost balance. One of the most pioneering strategies is FMT, currently recognized as the gold standard in the treatment of recurrent *C. difficile* infection: it has been observed that FMT could also be applied in the decolonization from vancomycin-resistant *Enterococci* [198]. Another important weapon is represented by probiotics, administered to reshape altered gut microbiota, prevent gut colonization by pathogen bacteria or counteract their presence [199]: they range from classical probiotics (*Lactobacillus*, *Bifidobacterium* and *Saccharomyces boulardii*), whose role is widely recognized (it has been demonstrated that a treatment based on *Lactobacillus* reduces the carriage of multi-drug resistant pathogens) [200], to next-generation probiotics (*Akkermansia muciniphila* and *Faecalibacterium prausnitzii*) whose potential still needs to be completely explored through additional studies but already represent futuristic tools for gut microbiota modulation in a personalized medicine perspective [201].

In this complex scenario, the modulation of the gut microbiota by antibiotics represents a pioneering challenge, only partially explored in the late years, from which we have still a lot to learn, with striking therapeutic opportunities even in non-infectious diseases. In this setting, antibiotics are aimed at harmonizing gut microbial function and, sometimes, composition; a more targeted and specific approach should be the goal to be pursued in the future, tailoring the treatment according to the type of microbiota modulation to be achieved and using combined strategies.

## Figures and Tables

**Table 1:** Antibiotic treatments in the main hepatologic benign disorder.

HEPATIC ENCEPHALOPATHY	SPONTANEOUS BACTERIAL PERITONITIS
*Rifaximin 1200 mg daily* → Secondary prophylaxis of hepatic encephalopathy (prevents OHE recurrence within 6 months from the first episode) in association with non-absorbable disaccharide; → Effective in the prevention of post-TIPS HE (starting the treatment 14 days before TIPS and continuing for about 6 months). Other effects of antibiotic prophylaxis with rifaximin: - Eubiotic modifications in gut microbiota; - Reduces bacterial virulence; - Reduces microbial translocation; - Increases serum levels of long-chain fatty acids; - Reduces serum proinflammatory cytokines.	*Prophylaxis* → Primary: rifaximin (1200 mg/day) better than Norfloxacin (400 mg/day); → Secondary: Norfloxacin and rifaximin both effective. Other effects of antibiotic prophylaxis: - Decreases plasma endotoxin; - Prevents bacterial translocation; - Modulates proinflammatory and anti-inflammatory cytokines; - Reduces the risk of death/transplant. *Empiric Antibiotic Treatment* → PMN > 250 cells/mm^3^ on ascites (do not delay waiting for bacterial culture results); → Regardless of PMN count, in cases of suspected SBP (presence of infection symptoms: fever, abdominal pain, unexplained HE). Antibiotic choice: → Community vs. nosocomial SBP; → Local bacterial resistance patterns; → Broad spectrum recommended (until susceptibility becomes available); → Third generation cephalosporins or amoxycillin/clavulanate or quinolones (ofloxacin and ciprofloxacin). Quinolones not indicated: → For the treatment of SBP developed in patients already on prophylaxis against SBP; → In those regions with high prevalence of quinolone-resistant microorganisms; → In cases of nosocomial SBP.

Abbreviations: HE = hepatic encephalopathy; OHE = overt hepatic encephalopathy; PMN = polymorphonucleates; SBP = spontaneous bacterial peritonitis; TIPS = transjugular intrahepatic portosystemic shunt.

**Table 2:** Characteristics and results of the studies about antibiotic treatment in patients with hepatic encephalopathy.

AUTHORS OF THE STUDY	STUDY DESIGN	STUDY POPULATION	AIM OF THE STUDY	PRIMARY ENDPOINTS	SECONDARY ENDPOINTS	RESULTS	
Glal et al. (2021) [57]	Randomized double-blind controlled clinical trial	60 cirrhotic patients	Efficacy and safety of 550 mg twice daily of rifaximin or 500 mg twice daily of Nitazoxanide for 24 weeks	Duration of remission, number of recurrent episodes, evaluation of HE-related clinical symptoms, serum levels of ammonia, TNF-α and octopamine and calculation of Chronic Liver Disease Questionnaire score	NA	Nitazoxanide (faced against rifaximin): → Statistically significant improvement in CHESS score and mental status. → A total of 136 days of remission vs. 67 days of remission for patients on rifaximin (P1 = 0.0001) and significant reduction in Child–Pugh score (P1 = 0.018). → Statistically significant decrease of serum ammonia, TNF-α, and octopamine levels. → Improvement in Chronic Liver Disease Questionnaire score.	
Bajaj et al. (2013) [48]	Interventional pilot study	20 cirrhotic patients with MHE	Analysis of the microbiome, metabolome and cognitive improvement after rifaximin	Evaluate the effect of rifaximin on the metabiome (determined by the interaction between phenome, microbiome and metabolome)	NA	Significant improvement in cognition (6/7 tests improved, *p* < 0.01) and endotoxemia (0.55 to 0.48 Eu/mL, *p* = 0.02). Increase of serum saturated and unsaturated fatty acids. No significant microbial changes were observed after rifaximin, apart from a modest decrease in Veillonellaceae and increase in Eubacteriaceae.	
Bass et al. (2010) [61]	Randomized, double-blind, placebo-controlled trial	299 cirrhotic patients in remission from recurrent HE	Efficacy and safety of 550 mg twice daily for 6 months in the prevention of HE	Time to the first breakthrough episode of HE	Time to the first hospitalization due to HE	In the rifaximin group: → A total of 31 of 140 patients (22.1%) and 73 of 159 (45.9%) in the placebo group (hazard ratio for a breakthrough episode in rifaximin group compared to placebo was 0.42; 95% confidence interval (CI), 0.28 to 0.64; *p* < 0.001), with a relative reduction in the risk of 58%. → For 19 of 140 (13.6%) patients: hospitalization involving HE, compared to 36 of 159 (22.6%) of placebo group (hazard ratio of 0.50 (95% CI, 0.29 to 0.87; *p* = 0.01), with a relative reduction in the risk of 50%. Incidence of adverse events: similar between the two groups.	
Eltawil et al. (2012) [62]	Systematic review of 12 studies	565 cirrhotic patients	Efficacy of rifaximin in the management of HE	Efficacy and safety of rifaximin for the treatment of patients with at least one episode of HE	Reduction of serum ammonia levels and changes in psychometric parameters (mental status, asterixis, electroencephalographic characteristics and HE sum) after treatment	Rifaximin group: → Clinically equivalent to disaccharides or other oral antibiotics (odds ratio (OR) 0.96; 95% CI: 0.94–4.08) but with a better safety profile (OR 0.27; 95% CI: 0.12–0.59). → Lower serum ammonia levels (weighted mean difference (WMD) = −10.65; 95% CI: −23.4–2.1; *p* = 0.10). → Better mental status (WMD = −0.24; 95% CI: −0.57–0.08; *p* = 0.15) and less asterixis (WMD −0.1; 95% CI: −0.26–0.07; *p* = 0.25) without reaching statistical significance. → Electroencephalographic response and grades of portosystemic encephalopathy presented better results in comparison to the control group (WMD = 0.21, 95% CI: −0.33–0.09, *p* = 0.0004; and WMD = −2.33, 95% CI: −2.68–1.98, *p* = 0.00001, respectively).	
Patel et al. (2022) [63]	2 Meta-analysis of 5 studies	(1) 555 cirrhotic patients (2) 784 cirrhotic patients	Safety and efficacy of rifaximin (1) over systemic antibiotics (oral quinolones) for SBP prevention (2) over placebo for SBP prevention	(1) Comparing rifaximin to systemic antibiotics for the prevention of SBP (2) Comparing rifaximin to placebo for the prevention of SBP	(1) Subgroup analysis comparing rifaximin to systemic antibiotics for primary and secondary SBP prophylaxis (2) Subgroup analysis comparing rifaximin to placebo for primary and secondary SBP prophylaxis	(1) Rifaximin: significantly more protective from SBP that systemic antibiotics (OR 0.38, 95% CI: 0.19–0.76, *p* = 0.01). OR for primary prophylaxis was 0.59 (95% CI: 0.32–1.09; *p* = 0.10). OR for secondary prophylaxis was 0.46 (95% CI: 0.09–2.29; p = 0.34). (2) OR for the development of SBP was significantly lower in patients receiving rifaximin compared to no antibiotics at 0.34 (95% CI: 0.11–0.99; *p* < 0.05). OR for primary prophylaxis was 0.53 (95% CI: 0.28–0.99; *p* = 0.05) in favour of rifaximin.	
Zuo et al. (2017) [64]	Open-label study	14 cirrhotic patients with MHE	Efficacy of rifaximin in restoring the gut microbiota of patients with MHE	To restore the gut microbiota towards the normal composition and functions	NA	After rifaximin: → Overall decrease in Chao1 index was detected subsequent to rifaximin, although with exceptions. → General decline in Shannon index was observed after rifaximin (predominantly in non-alcoholic patients).	Decrease in the abundance of Firmicutes (more apparent in non-alcoholic patients). Increase in 7 out of 14 patients of Proteobacteria. The remaining half showed unaltered or decreased abundance of Proteobacteria.
Kaji et al. (2017) [66]	Open-label study	20 patients with decompensated cirrhosis	Efficacy and safety of rifaximin 400 mg thrice a day for hepatic encephalopathy with the linkage of gut microbiome	To determine the efficacy of rifaximin for HE, evaluated with serum ammonia level, number connection test (NCT) and endotoxin activity	Effect of rifaximin on the gut microbiome	Rifaximin improves hyperammonia and cognitive impairment, with decreased endotoxin activity	Rifaximin did not alter the diversity and major components of gut microbiome, although the relative abundances of genus Veillonella and Streptococcus were lowered.
Kaji et al. (2020) [67]	Observational study	30 patients with decompensated cirrhosis	Efficacy of rifaximin 1200 mg/daily on intestinal permeability and gut microbiota	MHE symptoms and serum ammonia levels after 4-week rifaximin	Assessment of gut permeability with soluble CD163, soluble mannose receptor (sMR) and zonulin, after 4-week treatment with rifaximin. Assessment of gut microbiota with 16S rRNA gene sequencing, and serum pro-inflammatory cytokines after 4-week rifaximin treatment.	Improvement of MHE and lowering of mean serum ammonia levels (101.9 ± 30.9 µg/dL at baseline vs. 63.3 ± 19.4 µg/dL at RFX; *p* < 0.01). Serum levels of both sCD163 and sMR were markedly decreased by 4-week rifaximin treatment, while serum zonulin levels were unchanged.	No statistically significant differences in the richness (Chao1 index) (105.0 ± 38.5 at baseline vs. 92.1 ± 26.1 at RFX; *p* = 0.662) and complexity (Shannon index) (3.857 ± 0.642 at baseline vs. 3.727 ± 0.591; *p* = 0.776). 90 genera (58 Veillonella decreased significantly after rifaximin (*p* = 0.031) while the other genera unchanged. Rifaximin did not affect serum levels of TNF-α, IL-6, IFN-γ, and IL-10.

Abbreviations: HE = Hepatic Encephalopathy; MHE = Minimal Hepatic Encephalopathy; IFN-γ = Interferon-γ; IL-6 = Interleukin-6; IL-10 = Interleukin-10; NA = not applicable; NCT = number connection test; SBP = spontaneous bacterial peritonitis; TNF-α = Tumor Necrosis Factor-α; WMD = weighted mean difference.

**Table 3:** Characteristics and results of the studies about antibiotic treatment in patients with spontaneous bacterial peritonitis.

AUTHORS OF THE STUDY	STUDY DESIGN	STUDY POPULATION	AIM OF THE STUDY	PRIMARY ENDPOINTS	SECONDARY ENDPOINTS	RESULTS	
Kalambokis et al. (2012) [88]	4-weeks open-label, placebo-controlled, pilot study	16 cirrhotic patients with ascites and no history of SBP (CPS C)	Efficacy and safety of oral rifaximin 1200 mg daily	WBC, neutrophils and endoxotin levels in ascitic fluis at baseline and 4 weeks	Cytology of ascitic fluid and plasma endotoxin level at baseline and 4 weeks	Rifaximin group: WBC count (−40.00 from baseline, *p* = 0.004) Neutrophil count (−14,9 from baseline, *p* = 0.01) Plasma endotoxin (−1.7 from baseline, *p* = 0.03)	Placebo: WBC count (+11.00 from baseline *p* = NS) Neutrophil count (+3.3 from baseline *p* = NS) Plasma endotoxin (+0.1 from baseline *p* = NS)
Dănulescu et al. (2013) [89]	6 months case—control study	46 cirrhotic patients with refractory ascites (CPS C)	Safety and efficacy of rifaximin 1200 mg orally daily for SBP prophylaxis	Development of SBP within 6 months	Polymorpho-nucleates (PMN) count in ascitic fluid at 6 months	Rifaximin: One patient developed SBP A significant decreased of PMN was detected in ascitic fluid of 21 of 22 patients	Placebo: SBP was diagnosed in 4 patients An increase of PMN was detected in ascitic fluids of 14 patients
Hanouneh et al. (2012) [90]	Retrospective study	404 cirrhotic patients with ascites	Determine if rifaximin decreases the risk of SBP and improves transplant-free survival in cirrhotic patients with ascites.	Incidence of SBP during follow-up	Transplant-free survival rate	Rifaximin group: incidence rate of SBP was 0.09 per person-year 89% remained SBP free at 4.2 months with 72% SBP reduction in the rifaximin group (hazard ratio = 0.28; 95% confidence interval, 0.11–0.71; *p* = 0.007) Only 28.6% of patients expired, *p* = 0.045	Non-rifaximin group: incidence rate of SBP was 0.4 per person-year 68% remained SBP free at 4.2 months 43.7% of patients expired
Mostafa et al. (2015) [91]	6 months single blinded, randomized, case–control trial	70 cirrhotic patients with ascites	Safety and efficacy of rifaximin over Norfloxacin for the prevention of SBP	SBP rate after 3 months of therapy	Serum levels of Tumor Necrosis Factor-α (TNF-α), interleukin-6 (IL-6) and interleukin-10 (IL-10)	Rifaximin: No cases of SBP at 3 month Reduced levels of TNF-α and IL-6 (*p* < 0.05) Increased levels of IL-10 (*p* < 0.05)	Norfloxacin: Five cases of SBP at 3 months Reduced levels of TNF-α and IL-6 (*p* < 0.05) Increased levels of IL-10 (*p* < 0.05)
Sidhu et al. (2017) [92]	Systematic review of 5 studies	(1) 70 (2) 86 (3) 334 (4) 262	Efficacy of rifaximin versus Norfloxacin for the prevention of SBP occurrence/recurrence	Comparing rifaximin with Norfloxacin for SBP prevention of occurrence/recurrence	Mortality benefit with rifaximin as compared to norfloxacin Safety profile of rifaximin as compared to norfloxacin	All studies showed a reduced or equal incidence of SBP in the rifaximin group compared to norfloxacin group, although not always statistically significant	All studies showed a reduced mortality rate in the rifaximin group, although not always statistically significant. No serious adverse events were reported in any of thestudies with either of the drugs. Minor adverse events with similar with the 2 drugs.
Goel et al. (2017) [93]	2 meta-analyses of 5 studies	(1) 555 cirrhotic patients (2) 784 cirrhotic patients	Safety and efficacy of: (1) rifaximin over systemic antibiotics (oral quinolones) for the prevention of SBP (2) rifaximin over placebo for the prevention of SBP	(1) Comparing rifaximin to systemic antibiotics for prevention of SBP (2) Comparing rifaximin to placebo for prevention of SBP	(1) Subgroup analysis comparing rifaximin to systemic antibiotics for primary and secondary SBP prophylaxis (2) Subgroup analysis comparing rifaximin to placebo for primary and secondary SBP prophylaxis	Rifaximin was significantly more protective for SBP that systemic antibiotics (OR 0.38, 95% CI 0.19–0.76, *p* = 0.01). OR for primary prophylaxis was 0.59 (95% CI: 0.32–1.09; *p* = 0.10). OR for secondary prophylaxis was 0.46 (95% CI: 0.09–2.29; *p* = 0.34).	OR for the development of SBP was significantly lower in patients receiving rifaximin compared to no antibiotics at 0.34 (95% CI: 0.11–0.99; *p* < 0.05). OR for primary prophylaxis was 0.53 (95% CI: 0.28–0.99; *p* = 0.05) in favour of rifaximin.
Faust et al. (2020) [94]	Meta-analysis of 13 studies	1742 cirrhotic patients	Safety and efficacy of norfloxacin, ciprofloxacin, rifaximin, trimethoprim-sulphamethoxazole over plabebo for the prevention of SBP	Proportion of patients who developed SBP. Diagnosis was baased on a combination of clinical characteristics (fever and abdominal pain), cytologic criteria and ascitic fluid cultures	Risk of death or liver transplantation	All antibiotics were superior to placebo for secondary SBP prophylaxis with this rank: (1) rifaximin, (2) ciprofloxacin, (3) TMP-SMX, (4) norfloxacin and (5) placebo	The rank probability for efficacy of risk reduction of death, in ascending order, is (1) rifaximin, (2) ciprofloxacin, (3) norfloxacin, (4) TMP-SMX and (5) placebo.
Menshawy et al. (2019) [95]	Meta-analysis of 6 studies	973 cirrothic patients	Comparing safety and efficacy of rifaximin and norfloxacin over norfloxacin alone in the prevention of SBP	Prevention of SBP	Mortality rate, hepatorenal syndrome, septic shock, hepatic encephalopaty and GIT bleeding	Rifaximin and norfloxacin group had less incidence of SBP (RR 0.58, 95% CI [0.37, 0.92], *p* = 0.02) and hepatic encephalopathy (RR 0.38, 95% CI [0.17, 0.84], *p* = 0.02) compared to the norfloxacin group. No significant difference between rifaximin and norfloxacin in terms of frequency of SBP and success rate of primary prevention of SBP (RR 0.49, 95% CI [0.24, 1.01], *p* = 0.05; RR1.21, 95% CI [0.95, 1.55], *p* = 0.13, respectively).	
Assem et al. (2016) [96]	6 months open-label randomized case–control study	239 chirrotic patients with high SAAG (>1.1) ascites (CPS > 9)	Comparing safety and efficacy of norfloxacin and rifaximin vs. norfloxacin or rifaximin alone as primary prophylaxis for SBP	Development of SBP within 6 months	Overall mortality, incidence of infectious events, hepatorenal syndrome, liver transplantation and adverse event of drugs	Alternating norfloxacin and rifaximin determined lower probability to develop SBP in intention-to-treat (*p* = 0.016) and per protocol analysis (*p* = 0.039). No significant differences regarding the incidence or severity of adverse events and the incidence of HRS.	

Abbreviations: CPS = Child–Pugh Score; GIT= Gastrointestinal Tract; HRS = hepatorenal syndrome; IL-6 = interleukin-6; IL-10 = interleukin-10; OR = odds ratio; PMN = polymorphonucleates; SAAG = Serum-ascites albumin gradient; SBP = spontaneous bacterial peritonitis; TMP-SMX = trimethoprim-sulfamethoxazole, TNF-α = Tumor Necrosis Factor-α; WBC = white blood cells.

**Table 4:** Indications for antibiotics in acute pancreatitis.

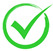 WHEN TO PRESCRIBE
Positive culture of necrotic material collected by FNA; Unequivocal imaging signs of infected necrosis (e.g., gas in peripancreatic collections); Proven bacteraemia; Associated biliary infection (e.g., cholangitis and cholecystitis); Clear worsening or no clinical improvement after 7–10 days of hospitalization according to SIRS indicators or APACHE II score.
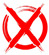 WHEN NOT TO PRESCRIBE
Mild interstitial pancreatitis; Prophylaxis of severe acute pancreatitis; Prophylaxis to prevent infection of sterile collections.
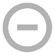 GRAY ZONE
Acute pancreatitis with elevated PCT; Pancreatic necrosis extention involving more than 50% of pancreas; Selective gut decontamination.

Abbreviations: APACHE II, acute physiology and chronic health evaluation; FNA, fine needle aspiration; PCT, procalcitonin; SIRS, systemic inflammatory response syndrome.

## Data Availability

Not applicable.
